# Situating support for people living with rarer forms of dementia

**DOI:** 10.1186/s12877-023-04268-4

**Published:** 2023-10-06

**Authors:** Mary Pat Sullivan, Paul M. Camic, Emma Harding, Joshua Stott, Gill Windle, Emilie V. Brotherhood, Adetola Grillo, Sebastian J. Crutch

**Affiliations:** 1 Faculty of Education and Professional Studies, School of Social Work, Nipissing University, North Bay, ON Canada; 2 Dementia Research Centre, Queen Square Institute of Neurology, University College London (UCL), London, UK; 3 Department of Clinical, Educational and Health Psychology, University College London (UCL), London, UK; 4 Ageing and Dementia @ Bangor, Dementia Services Development Centre (DSDC), School of Medical and Health Sciences, Bangor University, Bangor, UK

**Keywords:** Rarer dementias, Young onset dementia, Atypical dementia, Situational analysis, Care, Support

## Abstract

**Background:**

Awareness of a multitude of diseases that can cause neurodegenerative decline and their unique symptom profiles in the dementia care and support sectors remains limited. Obtaining an accurate diagnosis and post-diagnostic care and support is a challenge for many people and their families. As part of a larger study examining multi-component forms of support for people living with rarer dementias, the aim of this present study was to examine how rare dementia was situated within the complex social groupings, their organization and embedded discursive constructions that broadly form dementia care and support delivery.

**Methods:**

Adopting a situational analysis approach, we undertook an examination of public documents and organizational websites within the support sector for people living with dementia in Canada, England, and Wales. We also surveyed professionals to further explore the situation at the point of care and support delivery. Consistent with our approach, data collection and analysis occurred concurrently including the development of a series of analytic maps.

**Results:**

Recognizing the complexities within the situation, our findings provided new insights on the situated structures for support action and the discursive representations that illuminate both the limitations of the current support landscape and possibilities for a more flexible and tailored rare dementia support. Alongside, the predominant universal versus tailored support positionings within our data reinforced the complexity from which a promising new social space for people living with rarer dementias is being cultivated.

**Conclusions:**

The social worlds engaged in supportive action with people living with rare dementia are less visible within the shadow of a universally constructed dementia support milieu and appear to be negotiated within this powerful arena. However, their evolving organization and discursive constructions point to an emerging new social space for people living with rarer conditions.

**Supplementary Information:**

The online version contains supplementary material available at 10.1186/s12877-023-04268-4.

## Introduction

According to Harvey and colleagues [1], non-Alzheimer or vascular causes of dementia may account for up to 25% of all diagnoses. These atypical forms of dementia are also more likely to be diagnosed in people under the age of 65 years. Recently, Hendricks et al. [2] estimate that worldwide there are 3.9 million people between the ages of 30–60 living with dementia or what is commonly classified as ‘young onset dementia’. Whilst this classification, not unlike the unified term ‘dementia’, serves to identify dementia onset at a younger age, it provides no understanding of the characteristics of the condition or disease underlying neuro-cognitive decline.

Bio-medical recognition of the heterogeneity of symptoms and the underlying disease-causing dementia in both younger and older people have a long history. The nosological classification of Alzheimer’s disease (AD) by Emil Kraepelin in 1910, for example, was first used to distinguish the rarer pre-senile dementia from that which was affecting older people [3, 4]. Kraepelin’s contemporaries and many more since then have significantly advanced the aetiology and neuropathology of dementia to differentiate AD from its variants and other neurodegenerative diseases [4]. This medicalized lens, often criticized for reducing people to their set of symptoms, can though facilitate timely detection and clinical care options (e.g., pharmacological treatments) for people affected by lesser-known dementias such as behavioural variant frontotemporal dementia, semantic variant primary progressive aphasia, posterior cortical atrophy and others.

Not unlike other rare diseases, atypical forms of dementia often present unique challenges for individuals, families, and the care and support community. Previous research exploring different rare forms of dementia have shown that about 30% of people will receive an incorrect psychiatric diagnosis before a correct dementia diagnosis [5]. This is largely due to the range of cognitive (e.g., temporal and space disorientation, apraxia, aphasia, memory problems, deficit logical and abstract reasoning, acalculia, agnosia) and non-cognitive (e.g., apathy, anxiety, depression, irritability, disinhibition, hallucinations) symptoms that can present. For example, people living with frontotemporal dementia may struggle with recognizing and making sense of changing interpersonal relationships [6]. People living with posterior cortical atrophy can experience significant adjustments in their sense of independence and identity when confronted by profound difficulties with previously simple everyday tasks which rely on visual information such as reading, writing and driving [7]. For some, a new diagnosis may involve facing the possibility of genetic testing [8], navigating child or parental care responsibilities, employment loss, disability or benefits systems, and role changes within the marital and family systems (e.g., [9–11]). The transition from diagnosis to post-diagnostic support is also often delayed; and services, if available, often unsuitable [12, 13].

Undoubtedly, post-diagnostic care and support is positioned within existing and intersecting bio-medical, social, and political arrangements for people living with dementia (PLWD) more broadly. These arrangements, encompassing both systemic strengths and weaknesses, are translated to how the term ‘dementia’ is both used and understood, and to the various locations where direct care and support are delivered [14, 15]. For example, in many ways rare dementia does not escape the ‘rare disease paradox’ – the disease or condition causing the dementia is rarer than AD but the number of people who are collectively affected are many [16]. The public health ‘threat’ of large numbers of older people who will be diagnosed with dementia and ‘burdening’ existing health systems is also well documented [17]. Whilst the threat has largely stigmatized people living with dementia, it has successfully generated important research, policy and practice interest in dementia [17, 18]. The lower prevalence of rarer dementias, however, means people affected are fewer and scattered across populations, and with a lessened threat these conditions seemingly attract less attention within dementia discourses [19].

As part of a multi-site research study investigating the support needs of people affected by rarer forms of dementia and models for multi-component support [7], our interest was to examine how rare dementia is characterized and how support is constructed and organized within the extensive dementia care and support arena. Specifically, we wanted to develop an in-depth understanding of the tensions within the dynamic policy and care and support environments for PLWD, and the reported marginalization of people living with a rarer dementia (PLWrD). Using an iceberg metaphor, an exploration of what lies below the surface of direct clinical and support practice with PLWD will likely not directly inform a practitioner’s decision-making. However, the unseen structural elements which maintain the surface are integral to understanding the conditions for practice.  As Clarke and colleagues argue [25], *“If we want to understand what is going on in the situation, ‘the rest’ is part and parcel of it.”* (p. 362).

Rare dementia was defined as forms of dementia characterized by progressive difficulties with cognitive symptoms other than memory and/or occurring before the age of 65 [20]. Support was characterized as emotional, social, information or instrumental post-diagnostic interventions aimed at improving wellbeing for people living with rare dementia and their families [21, 22]. This differs from care, defined as services aimed at managing the progression of dementia including medication and post-diagnostic services provided by health providers such as psychological or speech-language therapies [23]. Our research questions were:

1. How and where are rare dementias and PLWrD made visible?
2. How is rare dementia positioned within the broader dementia discourses and what influences how it is understood?
3. How does current dementia discourse shape our understandings of rare dementia, the support needs of PLWrD and the organization of support?

## Method

### Approach

Recognizing that the experience of living with a rare dementia is positioned within broader and complex social environments, a situational analysis (SA) permitted an opportunity to problematize how ‘things came to be’ [24]. In its approach of conceptualizing the situation, SA moves beyond the examination of individual actors through the adoption of a wide angled lens to foreground the broader social, political, moral and cultural world, its processes, and relationships – its connectedness. In doing so, SA welcomes an attempt to make sense of social groupings, their relations, and from where action and discursive constructions originate and radiate [25]. Thus, SA draws together discourse and agency, action and structure, context, history, and the present moment, and their interaction with one another, to analyse the messiness of modern life or the gestalt of the situation of interest [25, 26].

Briefly, the origins of SA are Straussian grounded theory and build on Strauss’ assertion that all knowledge is situated – moving away from the simplification and organization of our social worlds to an acknowledgement of heterogeneity and complexity or ‘multiple truths’ [25]. Its theoretical underpinnings reflect its alignment with interpretivism and critical interactionism, and more recently also guided by positions within postmodernism, poststructuralism and posthumanism social theory. This is evidenced within its stance on knowledge development – *“if knowledge is to be productive instead of merely representative, we must be responsible to the kinds of worlds our knowledge practices enable and facilitate”* [25, p. 11] and a rejection of analytic certainty – and its methods aimed at revealing a bird’s eye view of the nexus of individual life and the broader and unstable social environment [27]. In this light, Clarke and colleagues [25] advocate for *“sensitizing concepts”*, or *“directions on which to look”* (p. 349) as suffice rather than the development of formal theory.

SA’s methods reflect its interest in unpacking or making visible the complexity and diversity of social worlds (i.e., loosely bounded groupings of people with shared perspectives, practices or norms), or the relational ecology of the situation [25], through cartography. The construction of a series of analytic maps (e.g., situational, social worlds/arenas and positional) constitute a process for thick analysis of the social features of the situation [28, 29]. Alongside the use of visual representations of the data are also other qualitative tools such as coding and memoing [27]. As far as we are aware, this is the first time situational analysis has been adopted to explore the dementia support landscape.

### Data collection and analysis

Messy and ordered situational maps were first completed to begin to identify the multiple elements within the rare dementia situation and their possible relationships, inform data collection, and facilitate our analytic work (Additional File 1 & 2). Recognizing the complexity of the situation itself and our interest in the broader structural influences that may shape the environment in which dementia support is understood and delivered, data was collected from a sample of discursive public documents and organizational websites of dementia charities, services, advocacy groups, research centres and other knowledge transfer groups in Canada (n = 53), England (n = 12), Wales (n = 14), United Kingdom (n = 11) (i.e., documents or groups relevant to all four devolved nations), and others with international oversight (n = 6). Data collection was initially purposeful, and access to documents or organizational websites often led to other sources not unlike snowball sampling. The national perspectives selected were those in which the researchers were situated and our interest in the organization of rare dementia support in these countries. Inclusion criteria involved consensus that each organization was recognized as providing health and/or social wellbeing information about dementia and/or direct services. Data was collected over an 18-month period, and documents or reports ranged from after the G8 Dementia Summit Declaration in December 2013 until January 2022 (Additional File 3). Using a bespoke data collection tool (e.g., organization/report, data extracts, memos), our purpose was to explore and select both content and discourses specific to rare or young onset dementia. Data collection and its discontinuation was an incremental process. Agreement on data saturation was determined when it appeared that new information did not appear to add significantly to our mapping development.

We also distributed an online survey to a range of professionals in the UK (n = 62) and Canada (n = 46) (Additional File 4) involved in service delivery (e.g., nurses, occupational therapists, speech-language therapists) during January to July 2021. The data collected from professionals permitted further understandings of the situation at the point of care and support delivery. In the UK professionals were accessed through the Rare Dementia Support, University College London,  membership and in Canada through social media. The survey consisted of 23 open and closed questions aimed at exploring practice with people living with specific types of rarer dementias (e.g., nature of support provision, adaptations to practice meeting unique needs, interagency working), and organizations/resources used to inform practice. We will report more on this survey elsewhere.

Like in grounded theory, data gathering and analysis were concurrent activities. The analytic steps began with inductive open coding, using an agreed but flexible coding framework, of content extracted from public documents and organizational websites, and survey responses by a team of four researchers. With analytic memoing, this process provided an opportunity to illuminate emerging external conditions (i.e., social groupings or structures), including the organization of perspectives – the who, what and how of supportive resources for people and emerging positionings (e.g., discursive sites and proposed action). Codes were then clustered into categories to further organize the developing groupings in the situation (e.g., older versus young onset, rare dementia, public health positions, human rights positions, dementia diversity, support offerings).

**Fig. 1:**
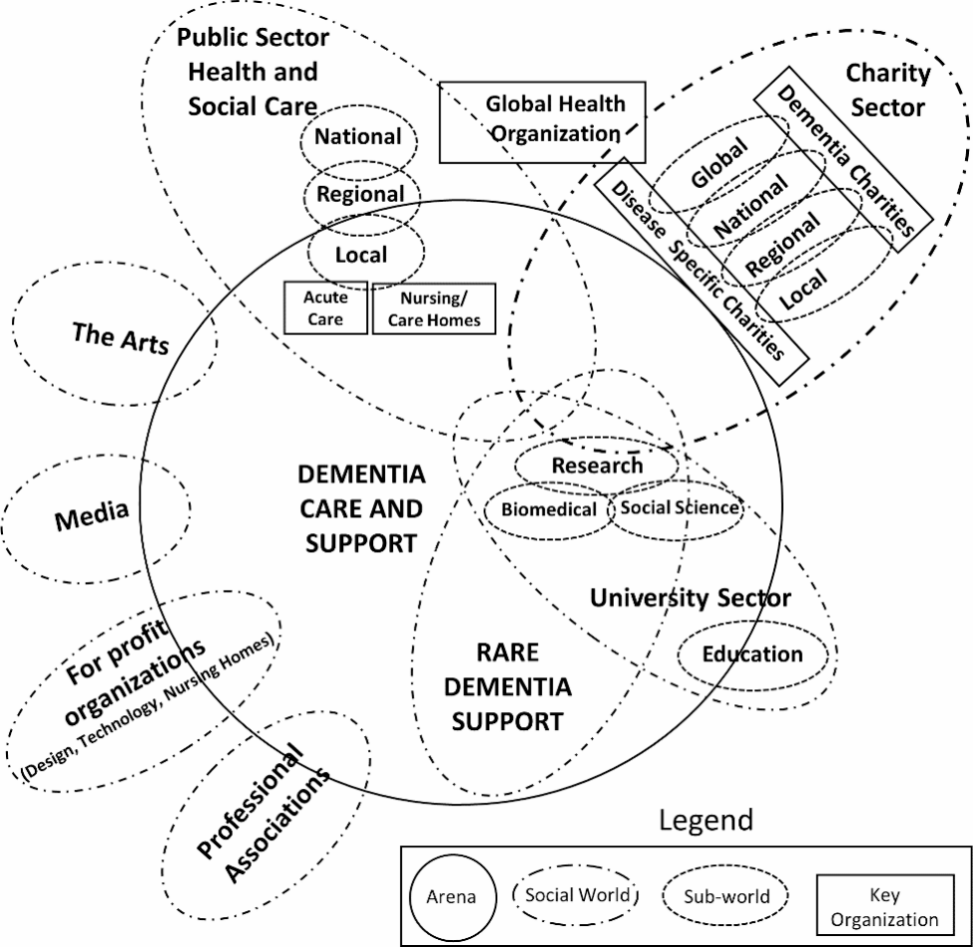
Rare Dementia Support: Social Worlds/Arenas Map

**Fig. 2:**
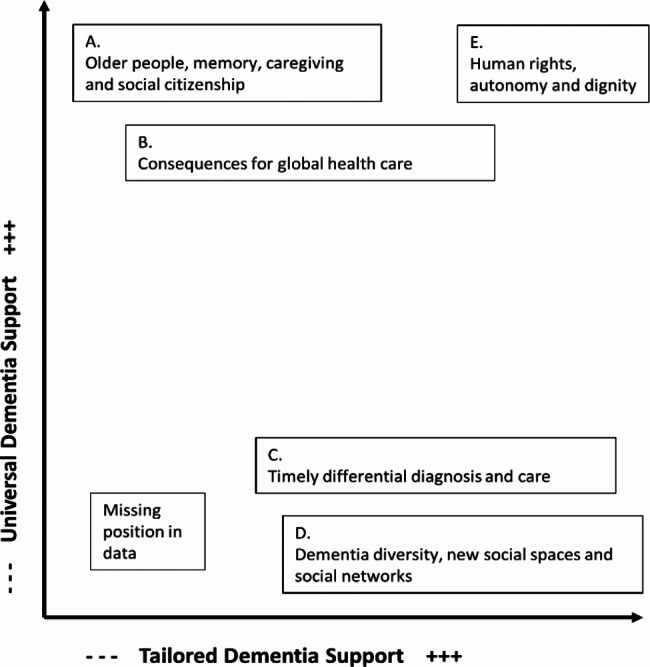
Rare Dementia Support: Positional Map

Further analytic map making by the research team was informed by, and interwoven with, our coded data. This progressive yet iterative approach involved several meetings and recorded for audio-memoing to inform mapping revisions which occurred up until and during the writing phase. The social worlds/arenas map laid out the actants and actors that form the situation and make collective sense of it (i.e., groupings where action takes place) (Fig. 1), and the final positional map completed the cartographic approach. The positional map provided an opportunity to visualize differing positions and/or controversies within the situation, as well as an articulation of the “silences” or spaces between positions (Fig. 2) [25]. The overall analytic process resembled Nicolini’s [30] ‘zooming in and zooming out’ whereby there was continuous back and forth between hypothesizing of the broader situation and its juncture with rare dementia support practices. Though a challenging approach, the systematic process of moving back and forth from data to map making helped to better conceive the messiness and begin to articulate some of the relationships between elements and discourses within the situation.

### Reflexivity

Reflexive engagement featured throughout the research process – from our original messy maps through to the writing stage. The messiness here, and our connected knowing (i.e., situatedness), was made visible during our frequent team meetings and our check-ins with the larger investigator group [31, 32]. We acknowledged our shared awareness that we too were situated actors and held positions as researchers funded to examine rare dementia, practitioners in this field, and some with direct personal experience supporting a family member with a rarer dementia. We were also simultaneously involved in other related projects where learnings came to bare on our understanding of the situation and how we came to construct it. Also relevant here, Clarke [24] has long argued that the hierarchical relationship between the researcher and the researched should be addressed by the researcher adopting the stance as a critical learner.

## Results

Our results provide an ecological and relational ‘big picture’ of the rare dementia support situation. The generation of understandings of how support for PLWrD is structured within the unpredictable terrains found in the social worlds of health and social care (e.g., government or charitable organizations) provide a glance into the provision of support at the level of the situation and its uncertainties. We first set out the layered social groupings that structurally situate rarer dementias, their connectedness, and make more apparent any visible collective commitments to social action (i.e., direct practice, education and training, advocacy) on behalf of and with PLWD (Fig. 1). We then present the identified positionings (e.g., topics or issues of focus for an organization such as person-centred dementia care or the importance of timely diagnosis), within these groups to begin to characterize shared ideology (e.g., values, viewpoints, loosely organized behaviour) as well as contrasting nonconformity which reinforces the complex ecology of the situation (Fig. 2). Thus, the structural orderings come to bear on what knowledge is both embedded and represented, and ultimately shaping the what and the how of support practices [33]. Our social worlds/arenas and positional maps also represent our tailored project maps to assist in the illustration of our results.

### Emerging from the shadows and the in-between dementia stories

#### International

The analysis of the international environment, including health-oriented and non-profit or charitable dementia organizations, provided some evidence of a significantly changed landscape for PLWD since the late 1970s when country-specific charitable efforts were organized to raise awareness and find a cure for AD. The next thirty years witnessed new global collaborations, the creation of worldwide federations and knowledge exchange forums for AD. Although these early global efforts were led by G7 countries, many of these initiatives now reflect the engagement of less industrialized states.

As set out in the data, with an increasing shift to health policy and global awareness leadership at the international level appeared to strengthen and then culminated around 2009 with key publications documenting the new public health priority called ‘dementia’. Targeted to various audiences, these reports provided evidence for dementia’s prevalence and its impact on individuals, families, and society at large. The focus remained that of older people with AD or dementia, and only cursory attention to rarer diseases or people affected at a younger age. Alongside, federated charities continued their mandate of awareness raising and generating funds for, primarily, bio-medical research. A more recent exception within the global leadership structure and appearing to be led by younger PLWD, demonstrated a more diverse voice of dementia. The organizational focus varied from the aforementioned with its compelling and distinctive emphasis on advocacy, human rights and maintaining quality of life while living with dementia.

Interestingly, the transition in language from AD to dementia appeared to coincide with neuropsychological advancements in characterizing AD progression, its variants, risk factors, and other neurodegenerative diseases also causing dementia [4]. The recognition of neuropsychological heterogeneity, as argued by Ballenger [18], also further politicized the dementia problem to reconstruct normal versus pathological ageing and encourage the dispersion of resources to fund research. Thus, the bio-medical research engine, often aligned with academic structures, seemed to confidently straddle both the public health and charity divisions as a focal point for funds to minimize the societal impact of the diseases causing dementia.

#### National

It was difficult to ascertain more fully the relationships among these global structures or federated networks and their direct impact at a more local level – particularly how care and support was organized and delivered due to the complex intersection among various social groupings within nations. However, the above distinctive yet complementary (sometimes overlapping) dictates were noted to be mirrored at national levels. This was evidenced in the country-specific dementia strategies and other health sector guidance, large national and government recognized charities with membership in the world theatre, professional associations, and universities connected to international forums curated by globally focused organizations. We also recognized the presence of the arts, the private sector and the media as additional social worlds, but determined it was more difficult to confidently establish their embeddedness in the rare dementia support situation.

The charity and university sectors seemingly dominated dementia support at this level. Whilst pre- and post-diagnosis care was available in the health sector, the contracted publicly funded health care did not appear to feature prominently among the intersecting structures for the provision of support. Rather the bio-medical emphasis here appeared to be differential diagnosis, indicators for treatment or other forms of care.

Among the charitable organisations at this level, there was a wide range of groupings. As mentioned earlier, most visible were the long-established and newer large charities with a leaning towards awareness, education and support provision for an older population with dementia. These larger and seemingly more powerful charities and their international counterparts were also a principal source of information on dementia for over half of the professionals surveyed. It was important to observe that these charities operated through fundraising initiatives, but they were also recipients of government funding. Here, rarer dementias or less imprecise ‘young onset dementia’ were acknowledged but seemed to be less frequently acted on in terms of support. For example, a tension noted in the data for one charity was the provision of specific bio-medical descriptions of the different types of dementia and a detailed report on young onset dementia but setting out resources presenting universal explanations for psychosocial support needs. These knowing and governing practices that collectivized PLWD were in contrast with other nationally led charities, condition specific or those focused on younger PLWD, with tailored messaging and reinforcing individualized practices [33].

Few and smaller in scope were disease specific charities or those led by PLWD. Unlike the larger dementia charities, they appeared to rely solely on public donations to deliver their primary activities including awareness, information and education, and in some instances, support. Professionals surveyed reported accessing a range of resources from these different charities and that they were appropriately tailored and helpful for practice.

In the data, the university grouping was distinctive given its various sub-worlds and interactions with other sectors, and its actions aimed at bio-medical and social science research (although recognizing these sub-worlds also involved other disciplines such as engineering and the humanities), knowledge mobilization, education, and including support delivery. Although presented at the national level, the social action emerging from some institutions was also visible at a local or regional level. As anticipated, this particular social world reflected what Clarke et al. [25] referred to as mavericks – individuals taking risks, innovating, or working against the grain of more typical dementia conventions such as that seen in the public sector or other charity worlds. Thus, activity production within smaller sub-worlds targeting novel support understandings for PLWD and PLWrD were apparent (e.g., the emergence of small support networks for atypical dementia that were also connected to knowledge development, service user led informational resources, designing objects for conversation or play, models of co-led applied research). How these unorthodox practices translated beyond this world was not easily discerned within the data.

Finally, there was little evidence of any professional associations with an engagement in rarer or young onset dementia from either an advocacy or educational stance. Some health-oriented associations, not unlike the health sector, gave explicit attention to dementia sub-types and best practices for diagnosis and care. For the allied health professions, however, rare dementia support did not feature within their professional oversight and regulation or educational mandates.

#### Regional and local

At a regional and local level there appeared to be a more dynamic arrangement among the various social worlds as the actors from these diverse groupings intersected with one another at the point of support delivery for PLWrD at home. Both the larger and smaller charity groupings played a key role in the delivery of supports, as did practitioners from the health and social care sectors. Here too, subworlds emerged as new local groupings formed to ‘transform’ support from collective orderings to holding younger PLWD in relation to others vis à vis an emphasis on inclusive activities and normality. Interestingly, ideologies here seemed to influence action which resembled that emerging from academic institutions.

The precise ordering at each regional or local level was not possible to establish. However, the findings from the practitioner surveys reinforced groupings outlined above and the navigation of these from a support practice perspective. For example, these practitioners openly acknowledged younger people living with dementia were ‘missing out’ or needed something different within the current organization of services:

*We have to oppose attitudes and policies that cause younger people to miss out on dementia services and try to disseminate information about rare dementias so medics and social workers have a better understanding.* (sUK31)

*Most people that I have supported who are living with a dementia who are younger are very aware of their age and don’t wish to join activities/clubs where the majority of attendees are older. I would love to link them to volunteer opportunities in the community not just within our organization as options are limited), social programs that are specific to their ages as well as support groups for them as well as for the care partners that are specific to the disease and their situations.* (sC27)

At this level there was more evidence of connections between the groupings through, for example, loosely formalized dementia networks or alliances and hyperlinks between organizational websites. These relationships appeared to demonstrate some of the fluidity of the boundaries of the distinct social worlds, the possibility of negotiations among the actors in these worlds, or dissention within the collective identity:

*I support people to maintain activities of daily living including engaging in meaningful activity and activity of choice…We run groups for people who are newly diagnosed with dementia and their carers including a one-off psychoeducational workshop and a 10 week…course aimed at those struggling to adjust to their dementia diagnosis. Neither of these groups are rare dementia specific though. We do work in partnership with our local Alzheimer’s Society who have just started running a group for those with younger onset dementia and can signpost people to this group.* (sUK20)

### Obscuring dementia diversity?

The ordering of support as described above guided what discourses or knowledge anchored the provision of direct support for PLWrD. We have arranged these in relationship to two continuums as set out in Fig. 2.

On the vertical axis we present universal or collectivist dementia support and some of the more dominant associated discursive constructions. On the horizontal axis we highlight tailored or individualistic support and the discursive constructions that were discrete from the aforementioned ones. Middle ground positions near each axis are also shown on the map. Consistent with the postmodern turn in SA, the positions do not represent specific individuals or groupings [25]. And whilst appearing as binary oppositions, they were layered and sometimes overlapped, and they may seem contradictory at times. Collectivist positions remain entangled with individualistic ones because the rare dementia support situation is embedded, connected, or part of the dementia care and support arena.

#### Positions a and B. older people, memory, caregiving and social citizenship

Consistent with both the historical development of care and support services for PLWD and the risk of dementia associated with advanced age, the leading messages framed dementia within the broader and alarmist global ageing discourses. With increasing longevity and the likelihood of frailty, dementia’s social impact on caregivers, families and communities, and the economic consequences for global health were not uncommon in the data. Progressive narratives of dementia were also evident, however, more recent discourses including those that describe disability, dependency, fear, or urgent need for action, still cast their shadow for anyone with a dementia diagnosis:

*If we truly want to overcome one of the greatest health issues of our time then we need a concerted effort across society; families, friends, employers and the Government need to wake up to what dementia clearly means…Dementia can be devastating. It can rob a person of their memories, their personality, and ultimately their life. There is no way to prevent it. It can’t be effectively treated. There is currently no cure.* (UK3)

The continuing and powerful portrayal of PLWD as victims – incapacitated and attributed the status of frail dependents – remained. What significance did a person who was older and who was robbed of memories, personality and *“ultimately their life”* have? Ascribed a new status within this discourse, PLWD occupied a social space characterized by insecurity and vulnerability (and burden) only relevant for *“management”* by others [17, 34].

The predominance of memory-led descriptions of dementia, or AD and impaired memory as a key diagnostic category suitably featured within the discourses on dementia care. Within support discourses a similar emphasis was observed and often reinforced memory as the early sign or only consequence of dementia-causing diseases. Although not always consistent, one organization provided recommendations on what to do following diagnosis of dementia:

*You have likely been worried and anxious about the changes you are seeing in yourself. Now that you have been diagnosed with Alzheimer’s disease, you may be concerned about the future. However, you have already taken an important first step in caring for yourself: getting a diagnosis.* (C2)

Acknowledging the above expertise, newly developed guidance to share understandings of dementia stated the following:

*You are encouraged to speak with your health care provider about memory loss when you become worried that it is impacting your day-to-day life. Having a few of these symptoms more than once in a while may be a sign of memory loss caused by dementia or another illness.* (C33)

PLWrD whose early symptoms are language difficulties, for example, are distanced from these support messages. Additionally, the cultural meanings associated with memory and remembering constructs may also be relevant here. Remembering is a highly valued human activity. It represents truth or, for some, the continuation of self. It connects us to one another. Practically, it supports independence and autonomy. A person with a failing memory at any stage of any condition is thus further disenfranchised, powerless, and in need of care – care that has likely been conditioned by these understandings [17, 35].

Within the frame of older people living with, most often, memory-led dementia, further evolving constructions were evident in different worlds. This evolution or shift did not appear to be fully re-positioned given dissonance within the discourse and the multiple social worlds generating these. How these constructions migrate to label PLWrD was difficult to determine. However, the absence of competing narratives was noted.

The universal dementia support discourses strongly featured understandings of caregiving by those who are often family members:

*You may not think of yourself as a carer, particularly if the person with dementia is a partner, parent or close friend. But both you and the person with dementia will need support to cope with the symptoms and changes in behaviour. It’s a good idea to*:

- *make sure you’re registered as a carer with your GP*
- *apply for a carer’s assessment*
- *check if you’re eligible for benefits*
- *find out about training courses that could help you…Caring for a partner, relative or close friend with dementia is demanding and can be stressful. It’s important to remember that your needs as a carer are as important as the person you’re caring for.* (E2.i)

Whilst meant to be supportive of those who provide both care and support, the universal caregiving discourse was layered with several possible meanings. First, with the persistence of neoliberal ideology and austerity policies there is a societal expectation of filial care to minimize the impact on publicly funded services [36]. And second, spouses, partners, sons and daughters, or friends have been labelled carer or caregiver, and in some ways, they too have become an object within the bureaucratization of their role [37] and needing management to fulfil their duties – *“All carers must have reasonable breaks from their caring role to enable them to maintain their capacity to care, and to have a life beyond caring.”* (W.2) The role is one that is confirmed as stressful and caregiver *“training”* to successfully perform the associated tasks will help mitigate the negative consequences – “*People living with dementia and their caregivers have access to education and training on dementia and available support services.”* (C19.i). However, this too fails to recognize that caregiving involves the more opaque emotional or relational work alongside illness-specific work and care tasks [38]. Also, the caregiver construction is that of one principal person in need of support and training to enable them to continue their role within the care dyad. These embedded meanings fail to notice, among other things, the impact of this new enforced identity, the diverse meanings or motivations for family and friendly care, and the complex arrangements for the provision of care and support [39, 40].

Finally, the support discourses across the social worlds largely reflected a trinity of meanings aimed at improving the lives of PLWD including person-centred care, the social model of disability, and social citizenship aligned with human rights. Overarching was an emphasis on addressing dementia stigma through advocacy and awareness raising. These meanings received emphasis in different social worlds and, unsurprisingly, a unified message or one that engaged older PLWD was difficult to identify. Principally observable at the international and national levels, how all of this was both understood and operationalized into direct or indirect support was also difficult to know:

*Enhance access to a range of person-centred, gender-sensitive, culturally appropriate and responsive services including liaison with local nongovernmental organizations and other stakeholders in order to provide information that empowers people with dementia to make informed choices and decisions about their care. Respect their rights and preferences and foster active collaboration between the person with dementia, their families and carers and service providers from the first symptoms through to the end of life.* (Int1.ii)

This shift in discourse is meaningful as the ‘dementia problem’ is relocated from the individual to the socio-political structures that have bearing on someone’s ability to maintain a good life despite cognitive decline [41]. Indeed, these abstract constructs may become more concrete in relation to how dementia-friendly initiatives are realized and perhaps distinct from both government and some charitable support services in promoting agency, supported autonomy and participation.

#### Positions C and D. Dementia diversity, new social spaces and social networks

Despite recognition within the bio-medical domain of neuropathological heterogeneity and some strong assertions for the need for specialist care [42], the continuing construction of dementia as forgetful older people bequeathed a blind spot in support discourses and representations of PLWrD. However, our data provided evidence of an emerging position (s) that addresses this gap. Critically, these alternative positions intersected with those presented above and were sometimes hidden within the universal approaches to support. Thus, this position may yet be less powerful. Though, the story of this still youthful rare dementia position was interesting given it emerged at the junction of the different social worlds, each with their own layered positions, and where these materialized to address care and support for PLWrD. This contrasting position was also more closely aligned to the bio-medical argument for timely differential diagnosis and care options – “*It is crucial to understand what lies behind the condition in order to help make sense of it and to know how to manage it.*” (UK12.i.)

In the diverse social worlds, atypical or young onset dementia was endorsed in the discourse. This was not unproblematic, however. For example, what constituted ‘rare’ varied, what diseases or conditions identified were inconsistent, and there was no uniform presentation of prevalence rates for either young onset dementia or specific diseases. In addition, the constructions in care discourse stressed the need for diagnosis-specific information, for example, “*providing a validated diagnosis of dementia subtype, including atypical presentations”* (E4). However, information and advice about ongoing *“management”* or support was found to be non-specific. For example, this same data source stated, *“Support should be person-centred and holistic, and it may be provided via health and social care, local authorities or voluntary organisations.”* Although valued for the ideals underpinning its definition (e.g., empowerment, participation), person-centred dementia care has long been criticized for being too ambiguous [43] and overlooking a multitude of dementia experiences [44]. Yet others have argued that it fails to address that ultimately a PLWD does so in relation to others including the state as social citizens, and in multiple interdependent and reciprocal connections with community, family, friends and others [45]. The promotion of person-centred approaches also fail to recognize a largely inflexible health and social care system [46].

Positions promoting the value of a novel social space for PLWrD were made visible through those social worlds, largely the new or smaller charities and university sector, that were critical of undefined or fragmented person-centred care and support. The emergence of these newer social worlds was also testament to a growing demand for something fresh. The following surveyed practitioner aptly described the relevance of a new social space by means of the age of consequence:

*Having flexible working hours to accommodate spouses who are working and school-aged children of those living with rare or young onset dementias. It’s very important to set the stage of dignity and respect for any conversations that are had with couples and families so that all feel that their voices are heard. It’s also important to recognize that the issues affecting those living with rare or young onset dementias and their families are often quite different due to the ages of those affected and the subsequent losses they experience, e.g., haven’t had a chance to experience retirement dreams that were planned; loss of role of parent at a critical time in the young person’s life (pre-teen, teen years), etc.* (sC27)

As demonstrated here, the age of consequence was amplified by references to family, including children and parenting roles, employment and “retirement dreams”. And as reinforced by others (e.g., [47, 48], this was a characteristic that was not evident within the universal positions. Thus, this distinctive space and its psychosocial needs reinforced constructs associated with a tailored family and/or friend system support.

This emerging tailored position was strengthened with an additional focus on the necessity of disease-specific information – *“For how common it is, Lewy Body Dementia (LBD) is almost completely unknown. Those touched by its diagnosis have rarely heard of it beforehand. It is also under-diagnosed, misdiagnosed, and misunderstood: these realities lead to needless, tragic outcomes”* (C45.i) *–* and imagining a social space distinct from the more dominant one. Messages of infirmity evident in the universal positions (and support structures) were challenged and instead foregrounded ‘self’ and ‘living’, notwithstanding living with dementia:

*To get through this you need to take charge of your life again. Build up your confidence, take up all the things you did before. Don’t allow yourself to be consumed by dementia. I can still do many things I did before. Some I need a little help with, and if so, I will ask. Be as independent as you can. Don’t let people undermine you, to fit someone’s tick box.* (UK23)

Despite the apparent emphasis on individual responsibility for living well with dementia, the value of a new (normalized) social space, a new dementia identity, for the increasingly recognized number of people affected by rare or young onset dementia is not uncommon in the growing body of literature addressing this population (e.g., [7, 47]). The position was also persuasively presented here:

*In connecting with* [organization], *you’ve found a community of people who understand, and a source of information, resources, help, and hope for a better future. Support, connection and the latest information on managing FTD are all available here. You don’t have to face this journey alone*. *By staying connected with* [organisation], *you’ll have a knowledgeable partner – and a reliable source of help and information – by your side every step of the way.* (C41.i)

Social connection, including the value of the maintenance of meaningful relationships, was also a strong feature within this position. These connections appeared to define and distinguish this new social space. Social bonding included the maintenance of existing connections (e.g., family and friendships), and new social networks including peers living with similar conditions. For example, one organization promoted ‘active living’ with its key program outcomes as: *“individual role in managing health; timely, age-appropriate support; meaningful community participation; equal access to physical activity; friendships and camaraderie; and family-oriented, consistent resource.”* (C.20i). Even more influential was an emphasis on peer connection:

*… our online community, can help people with less common types of dementia to feel less isolated. If you’re diagnosed with one of the less common causes of dementia, it may be harder to find people who understand the specifics of what you’re going through. If they’re mostly affected by Alzheimer’s, vascular dementia or both – even if they can relate to many aspects of your experiences – you might also want to connect with people who know what it’s like to live with your particular condition.* (E1.i)

New bio-social groupings at the local, national and international levels filtered disease or condition specific information, education and other forms of support. As well, these groupings made visible dementia diversity and, for some, seemed to give people agency to combat dementia stigma or advocate for new forms of dementia support. These more recent bio-social groupings demonstrated further incongruence with the universal dementia support discourses.

#### Position E. Human rights, autonomy and dignity

The ideological positionings on human rights, autonomy and dignity are now more familiar within dementia discourse and were apparent within our data as indicated above. The messiness of the situation was especially apparent here. The right to receive support, assisted autonomy or interdependence, and living with dementia free from discrimination was shared across the universalistic and tailored positions. However, sundry conceptualizations of citizenship resulted in discursive tensions resulting from how people living with dementia were portrayed (e.g., powerless versus active agents), in what manner rights or autonomy were conveyed, and how these possibly shaped care and support systems. Nevertheless, this emphasis on citizenship in either position inferred that people affected had the right to services within an environment that challenged the social devaluation of PLWD. An excerpt from one national charter of rights for PLWD used a strengths-based approach to facilitate capabilities through meeting need, participation, growth and agency. Although the conditions necessary for action were left undeveloped as in this statement:

*To access support so that I can live as independently as possible and be as engaged as possible in my community. This helps me*:

- *Meet my physical, cognitive, social, and spiritual needs*,
- *get involved in community and civic opportunities, and*
- *Access opportunities for lifelong learning.* (C2.i)

Increasing evidence of rights related discourse was apparent in various social worlds – particularly those led by people affected by dementia, and advocating for a person’s right to information and support was also evident in the professional survey data, for example:

*…it is usually me who tries to support people in employment and keep this going for as long as possible, and sometimes I have to advocate for people when I feel employers are breaching their rights. The same goes for statutory services not being provided when the Care Act says they should be. I write a lot of letters.* (sUK21)

Although citizenship approaches are premised on the notion of self-cognisance and fail to recognize how the abstract is realized (or not) at the point of service delivery [44], an emphasis on relational spaces within the tailored position appeared to provide a distinctive understanding. Relational social spaces or social bonding provided opportunity for everyday expressions of citizenship through relationships. Social networks facilitated citizenship practices [41]. Here too though, consideration of the trajectory of the disease and relational citizenship in the later stages of living with dementia was not evident in the data, although this has been developed in Kontos and colleague’s [45] concept of relational citizenship. Also silent within the data was the absence of specific attention to human rights and diverse populations such as newcomers, LGBTQ individuals, or those living in care homes.

## Discussion

The purpose of our study was to explore how rare dementia support was understood or characterized and organized within the dementia care and support arena. Our interest was motivated by repeated reports in the literature that people affected by rarer or young onset dementia were marginalized owing to complex trajectories through both the medical and support sectors. Returning to our specific research questions, we were interested in how and where rare dementias and PLWrD were made visible and positioned within dementia discourses. In addition, we were concerned with how the emerging discourse shaped the support needs of PLWrD and the subsequent organization of support. Our data, collected through discursive documents and a survey of professionals, and analyzed through a series of maps provided a complex bird’s eye view of support for PLWrD embedded within and alongside dementia care and support structures more broadly. The constitutive elements and related processes within the full situation were difficult to untangle owing to PLWrD situated with the larger dementia care and support arena. Yet our examination of the ecology of the situation permitted an in-depth gaze at where the issues for rare dementia support were negotiated and where the social lives of PLWrD were ultimately organized.

Recognizing both the complexities or messiness within the situation, our understandings provided new insights on the situated structures or conditions for support action and the discursive representations that illuminate possibilities for a fluid rare dementia support landscape. Our exploration of the layered social groupings relevant to supporting PLWrD provided clarity regarding both discursive sites and the sites of power. At the international, national, regional and local levels, dementia support was primarily organized and led by social groupings taking action for older people living with memory-led conditions despite the bio-medical recognition of dementia diversity. Here too though, the importance of an accurate assessment and diagnosis was reinforced (e.g., published dementia care pathways). Yet this diagnostic tailored approach did not confidently translate to tailored post-diagnostic information and support. And ultimately, this lack of recognition conceivably maintained a universal organization of government funded support. The absence of action for PLWrD is consistent with other empirical studies exploring support for people living with rare or young onset dementia (e.g., [12], [13]).

We identified that rare dementia support was predominantly made visible through the discourses and action of new and smaller charities, including some co-produced by people affected by rare or young onset dementia. Here, awareness, socialization, support and tailored information for the type of dementia characterized their social action. In absence of government recognition and funding, however, these supports are no doubt severely impacted by the non-profit starvation cycle, competition with the larger government recognized charity sector for public donations, and a streamlined infrastructure limiting their ability to engage beyond their immediate support role. Their long-term sustainability remains unknown. Significantly, the university sector also strongly featured here as their engagement in knowledge production involved new and varied dementia narratives and novel support development. Their intersections with other social worlds were apparent, although their research and translational practice reach (influence) into the broader support arena was not fully apparent.

Five intersecting discursive positions were anchored within this organization of support – reinforcing the structural influences on the lives of people affected by dementia. Given organizational leaders were primarily structured to reduce the economic threat of an aging population, the discursive constructions were powerful ones and seemingly conditioned support approaches. Vulnerable older people with memory problems and their caregiver, or the ‘dementia problem’, became constructs for management within the bureaucratization of support. Universal approaches to support were constructed under representations of, primarily, person-centred care and social citizenship, despite lack of clarity on how these ideals translated to practice.

This dementia social space was transformed with the emergence of more nuanced constructions of dementia by and for people living with rare or young onset dementia. A demand for disease-specific information coupled with the age of consequence resulted in a new social space or new dementia identity that was primarily relational. Social connection through new bio-social groupings characterized this tailored approach – and an approach to human rights or citizenship practices that were also relational. Familiar in more recent literature on young onset dementia and increasing demands for support innovation (e.g., [7, 47]), this new story is less developed and unfinished.

### Methodological considerations and limitations

SA permitted a novel approach to exploring the rare dementia support situation. It encouraged the introduction of a critical and unrestricted lens to make visible the current situatedness of rare dementia support. It also facilitated a thorough consideration of the complexity that appears to hinder social action and limited progress across and within the care and support sectors to address dementia diversity among PLWD. We recognize that the rare dementia support situation is also made visible through the voices of people with lived experience. Our work indirectly brings those voices here through the organizations led by people affected by dementia that were reviewed and members of our research team. Although adding to the complexity of the SA approach, our analysis may be enriched by a future project that merges our extensive in-depth interviews with PLWrD conducted as another work package in our larger study. Representations of social activities through, for example, observations of direct practice may also provide additional insights into the situation.

The process of conducting a SA was challenging due to the enormity and ‘messiness’ of the situation itself and this was overwhelming at times. Equally demanding were repeated cycles of data collection, analysis, memoing, and ‘staying with it’ as our understandings continually took shape. The repeated engagement with our larger interdisciplinary research team, each with diverse perspectives on supporting PLWrD, supported this process and provided reassurance when the very natural tendency to try and put the situation in order surfaced. Multiple views during mapping process were also highly valued.

We recognize that any situation is evolving on a continuing basis. Thus, our findings have temporal limitations and need to be understood within a particular moment in time. We also recognize that our SA is limited within the scope of the countries examined, and each with similar yet different health and social care systems. We also acknowledge that as the situation changed, organizations and/or documents may have gone unnoticed. Additional data such as historical records and/or interviews with key opinion leaders within the social worlds/arena may also have contributed to a more detailed analysis of what specifically makes up rare dementia support, including for those from diverse populations.

### Implications for practice and research

Our findings suggest relevant implications for both practice and research. The ecology of the situation is becoming increasingly complex due to the number of social worlds and sub-worlds engaged in supporting PLWD, our evolving discursive constructions about living with dementia (e.g., living well versus suffering with), bio-medical advancements recognizing types of dementia, and population diversity. The contraction of publicly funded services and the expansion of charities struggling for loyal donors have further contributed to this unstable environment. Those on the front-line of practice do or do not recognize and can or cannot navigate the consequences. Despite the layering and intersection of the various dementia care and support groupings their less flexible boundaries may be inhibiting the bridging necessary to strengthen tailored action for PLWrD. This is particularly relevant for those interested in, for example, service growth or innovation and ensuring the unfamiliar and varied voices of lived experience lead to effective and equitable support. Capacity building within each of the social worlds is also relevant. From a research perspective, our study points to strengthening rare or young onset dementia translational research and knowledge mobilization as another necessary bridge for transforming the situation with and on behalf of PLWrD.

## Conclusion

The rare dementia support situation is a complex one layered among numerous mature and newer social groupings, relationships and discourses within the dementia care and support arena. SA permitted an alternative method to explore how the support situation for PLWrD may have come to be through our examination of policy, government and charitable organizational discourse, and the experience of practitioners working with PLWD and PLWrD. Namely, we cast an unconventional eye on how the conditions for support practices are anchored within intersecting international, national and local social worlds of care, support, education, advocacy and research. Within a predominantly collectivist position, supportive action with PLWrD was less visible within the shadow of powerful universally constructed dementia representations. Thus, a largely bureaucratized and inflexible support milieu nurtured the marginalization PLWD who do not fit what is familiar. However, the evolving nature of these social worlds and sub-worlds and their distinct discursive constructions pointed to newer representations of PLWD and provide evidence of an emerging and contrasting social space for PLWrD – but one that is still situated.

### Electronic supplementary material

Below is the link to the electronic supplementary material.

**Additional File 1**: Messy Map

**Additional File 2**: Ordered Situational Map

**Additional File 3**: Discursive Public Documents

**Additional File 4: Table S1**: Sample characteristics for Blinded (UK) practitioner members (N = 62). **Table S2**: Sample characteristics for Canadian practitioners (N = 46). **Table S3**: Frequency of work with people affected by rare dementias

## Data Availability

The documents and websites that support the findings of the study are available to the public online. The full datasets analyzed in this study are available from the corresponding author on reasonable request.

## Abbreviations

AD: Alzheimer’s disease
PLWD: Person living with dementia
PLWrD: Person living with rare dementia
SA: Situational analysis
