# Supplementary material for: Situating support for people living with rarer forms of dementia
Source: BMC Geriatr. 2023 Oct 6;23:627. doi: 10.1186/s12877-023-04268-4 (PMC10557369; doi:10.1186/s12877-023-04268-4)
Supplement: Supplementary file 1 — Additional File 1: Messy Map [file 12877_2023_4268_MOESM1_ESM.docx]

Advocacy

Professional bodies

Dementia threat and burden

Support group members

Change

Language

Training and education

Treatment

**RARE DEMENTIA SUPPORT**

Dementia Strategy

Employment sector

Liking for group vs individual support

Additional File 1: Messy Map

Maximise what’s available

Support outside of group

Positive risk taking

What happens in and after group

Scientists

Charities

Support structure

Disease process/stages

Genes

Funding sources

Signposting

Prevention

Fundraising

Neighbours

Dementia stigma

Universities & Colleges

Directive

Technology as solution

Directive

Cost of groups

WHO

Knowledge sharing

Group facilitators

Faith

Misdiagnosis

Austerity governments

Rare dementia support

Urban vs rural

Citizenship

International dementia organisations

Normalisation

Public health agencies

Digital resources

Hope & hopeless

Dementia celebrities

Researchers

Diversity

Research Funders

Relationships

Utility of support

Medical model

Volunteers

Information

Referral sources

PM’s Dementia 2020 Challenge

People living with dementia

Experience of “isms”

Changing status

Rare

Identity

Rights agenda

Carers

Dementia type

Political landscape

Arts & cultures

Agency

Social connection

Individualised patient care

Navigation

In-person vs virtual support options

Peer support

Age

Support group guest speakers

Social benefits

Dementia Friendly

Gatekeepers

Socioeconomic status

Friendship

Community as solution

Alzheimer’s Societies

Stigma

Reciprocity

Health care system

Friends

Aesthetics of support venues

Geography

Access

Centres of Excellence

Community as a Solution

Social care

Students

Positive identity

Shock & adjustment

Difficulties accessing support

G8 Summit

Families

Religious support

History of Alzheimer’s movement

Life course

Hold the key to real understanding & needs

One-to-one support

Branding

Volunteers

New pharma treatments

Awareness campaigns

Neurology

Practical tips

Navigating services

Capacity building

Investment in dementia care & research

Emotions

Evidence-based medicine and care

Individual responsibilisation

Lived experience

Clinicians

Care vs cure

Involved

Allied health

Group expectations

Location & transport

Empowerment
