# Supplementary material for: Situating support for people living with rarer forms of dementia
Source: BMC Geriatr. 2023 Oct 6;23:627. doi: 10.1186/s12877-023-04268-4 (PMC10557369; doi:10.1186/s12877-023-04268-4)
Supplement: Supplementary file 2 — Additional File 2: Ordered Situational Map [file 12877_2023_4268_MOESM2_ESM.docx]

Additional File 2: Ordered Situational Map

| Individual Human Elements/Actors | Non-human Elements/Actants |
| --- | --- |
| People living with dementia (PLWD) | Transport |
| Caregivers | Venue environment |
| Parents | Data protection regulations and consequences |
| Children at home | Digital resources |
| Facilitators | Personal finances |
| Doctors | Home environment (physical) |
| Health and social care professionals (e.g., support workers, dementia advisors, social workers, care navigators, occupational therapists, nurses) | Accessibility of support group venue |
| Friends | Educational (informal and formal) resources |
| Neighbours | Resources/other communications |
| Politicians/policy makers in government(s) | Time |
| Researchers | Information about the disease |
| Educators (not only professionals) | Name, branding, positioning of groups |
| All those we never meet (PWD/carers) | Leaflets, newsletters, other print resources |
| Other support group participants such as volunteers and guest speaker (s) |  |
|  |  |
| Collective human elements/Actors | **Implicated: Silent Actors/Actants/Non-human elements** |
| Support groups—different types | Goals and aims of sessions |
| Alzheimer’s Societ(ies), AgeUK (or equivalent) | Label of a rare dementia |
| Health and social care organisations | Other members of the PLWD family/friendly network |
| General public | Dementia care community |
| Developing new relationships | Dementia research community |
| Loss of relationships | Medical community |
| Universities and research teams |  |
| Organisations support groups are attached too/based in (e.g., hospital, charity) |  |
| Living situation/household/family make up |  |
| Importance of ‘group’ |  |
|  |  |
| Discursive Constructions of Individual and/or Collective Actors | **Discursive Constructions of Nonhuman Actants** |
| What does it mean to have a rare dementia? | How does the home as a place support/inhibit? |
| Expectations from groups? Assumptions/preconceived ideas about groups? | Culture |
| Experiences of caring for someone with a rare dementia | Faith/spirituality (conflict with religious days) |
| Awareness of rare dementia | Transfer of knowledge/knowledge exchange |
| Concerns about safety of PLWD | Technology (digital resources) as a solution |
| Sharing within groups (e.g., advice, guidance, emotional, tips/suggestions) | Individualized medicine |
| Mini-collective attitudes within groups (e.g., respect, encouragement, acceptance, normalising) | Support groups as a solution |
| Stigma |  |
| Hopelessness |  |
| Living well with dementia |  |
| Advocacy (e.g., family members, professionals, others) |  |
| Transfer of knowledge/knowledge exchange |  |
|  |  |
| Political & Economic Elements | **Sociocultural/Symbolic Elements** |
| Local, regional and national policies (e.g., age-based funding) | Media representations (e.g., Dementia ‘stars’ like Barbara Windsor, Glen Campbell) |
| Enactment of linking of local, regional and national policies |  |
| Resources needed to run groups (human, non-human) | Who attends support groups? (e.g., ethnicity) |
| Research (including methodologies specific to this project, evidence-based support) |  |
| Public and private funding |  |
| Power in terms of who decides what to fund |  |
| Hospitals and universities |  |
| Peer support is cheap |  |
|  |  |
| Temporal Elements | **Spatial Elements** |
| Diagnostic journey (e.g., first symptoms, adjustments, misdiagnosis, specificity) | Going from home to a group session |
| Discovering/ being referred to a RDS group | After a support meeting what happens (home, shopping, tea)? |
| What happens during a group session? | What happens when leaving home becomes too arduous? |
| In addition to groups, what does support look like over time? | Geographical location |
| Length and/or stage of illness and corresponding needs | Other sources of support nearby (e.g. postcode lottery) |
| Bereavement | Support group setting |
| Time of life (e.g., employment status, family position, existing care commitments) | Virtual/online access (live streaming vs. pre-recorded) |
| Signposting/onwards referral to other organisations/services | (within UK) South of England dominance (regional variations) |
| History of dementia care (e.g., Alzheimer Society, dementia strategy) and research agendas |  |
| Progressive/degenerative nature of conditions |  |
| Care transitions (hospital/home care) |  |
|  |  |
| Major Issues/Debates (Contested?) | **Related Discourses (historical, narrative, visual)** |
| Rare dementia support within a limited resource society-should we be specialising? | Barriers and facilitators for groups |
| Within support groups, what is and is not discussed? | What is the life of a group—how does it change over time? (What sustains it? What threatens it?) |
| Personal challenges for caregivers (burden of care) | Research methodologies |
| What “power” /agency resides in groups? | Public discourses (e.g., dementia friendly communities, living well with dementia, active ageing, ageing population, ‘tsunami’ of dementia, dementia as memory loss, burden of care) |
| Is rare dementia support only for white middle class people? |  |
| Role of researchers, methodology, epistemological and ontological debates, outcomes, different agendas | **Other Key Elements** |
| Threats to groups and the entire rare dementia support ‘system’ | Facilitating care planning (groups, health and social care, financial planning) |
| Differences of perspectives within families | What are the components of *being* “supported” (how is this understood, what does it feel like)? |
| Care vs. cure (an ongoing debate) | What are the formats and structures of groups? |
| Withdrawal of state from provision of services (i.e., responsibilisation) | What are the components of a group? (micro) (e.g., information sharing, connection, normalising) |
| How do international and national organisations affect/impact rare dementia support? | Types of groups (i.e., specific diagnosis, mixed, general) |
| WHO | Acceptance of diagnosis |
| Alzheimer Disease International | Information about the disease |
| Dementia Alliance | Gender |
| World Dementia Council | Sexuality |
|  | Cultural/ethnic influences/language differences |
|  | Premorbid (i.e., life before problems began) |
|  | Personalities of PLWD and carer |
|  | Identity |
|  | Type of rare dementia and symptoms |
|  | Key events (e.g., care home/hospital admission, telling children) |
|  | Benefits of groups |
|  | Comorbidities |
