# Supplementary material for: Situating support for people living with rarer forms of dementia
Source: BMC Geriatr. 2023 Oct 6;23:627. doi: 10.1186/s12877-023-04268-4 (PMC10557369; doi:10.1186/s12877-023-04268-4)
Supplement: Supplementary file 3 — Additional File 3: Discursive Public Documents [file 12877_2023_4268_MOESM3_ESM.docx]

Additional File 3: Discursive Public Documents

|  | **Document** | **Organization** | **Year** | **Primary Audience** |
| --- | --- | --- | --- | --- |
| **INTERNATIONAL**  **(Int)** | | | | |
| 1. WHO  [www.who.int](http://www.who.int)  *“The World Health Organization is the United Nations agency dedicated to global health and safety. The Organization connects nations, partners and communities to promote health and serve the vulnerable.”* | 1. Website 2. Report: Global Action Plan 3. Report: iSupport for Dementia 4. Report: mhGap 5. Report: Global Dementia Observatory 6. Educational module 7. Report: Risk Reduction of Cognitive Decline 8. Report: Towards Dementia Inclusive Society 9. Report: Synthesis Report 67 10. Report: Help for Care Partners (with ADI) | Intergovernmental | 2020-22^[[1]](#footnote-1)^  2017  2019  2019  2018  n.d.^[[2]](#footnote-2)^  2019  2021  n.d.  2016 | General Public, Professionals |
| 2. UN  [www.un.org](http://www.un.org)  *“The United Nations is an international organization…made up of 193 member states…can gather together, discuss common problems, and find shared solutions that benefit all of humanity.”* | 1. Website | Intergovernmental | 2020-22 | General Public |
| 3. ADI  [www.alzint.org](http://www.alzint.org)  *“We work by empowering Alzheimer and dementia associations to offer care and support for people with dementia and their care partners. Globally, we strive to focus attention on dementia, maintain it as a global health priority, and encourage investment in research.”* | 1. Website | Non-Profit | 2020-22 | General Public |
| 4. Alzheimer Europe  [www.alzheimer-europe.org](http://www.alzheimer-europe.org)  *“Alzheimer Europe is the umbrella organization of 37 national Alzheimer’s associations from 33 European countries. Our mission is to change perceptions, policy and practice in order to improve the lives of people affected by dementia. We are a non-profit non-governmental organization and will achieve our mission by providing a voice to people with dementia and their carers, making dementia a European priority, changing perceptions and combating stigma, raising awareness of brain health and prevention, strengthening the European dementia movement and support dementia research.”* | 1. Website | Non-Profit | 2020-22 | General Public |
| 5. World Dementia Council  [www.worlddementiacouncil.org](http://www.worlddementiacouncil.org)  *“…an international charity established by the G8 (now G7) at the London Dementia Summit in 2013. At the London summit the G8 made historic commitments to improve the lives of people affected by dementia today and to speed up the development of disease modifying drugs so that the first treatment is available by 2025.”* | 1. Website | Non-Profit | 2020-22 | General Public |
| 6. Dementia Alliance International  [www.dementiaallianceinternational.org](http://www.dementiaallianceinternational.org)  *“DAI is a collaboration of like-minded individuals diagnosed with dementia providing a unified voice of strength, advocacy, and support in the fight for individual autonomy for people with dementia.”* | 1. Website | Non-Profit | 2020-22 | General Public |
| 7. G8 Summit Declaration  <https://www.gov.uk/government/publications/g8-dementia-summit-agreements/g8-dementia-summit-declaration>  *“We will continue our efforts to work together in line with the commitments in this Declaration and Communiqué, but we recognise that dementia is an issue which affects people in countries throughout the world. Consequently, we encourage all countries and multilateral organisations to come together and take action to reduce the risk to health and to economic development which dementia currently presents.”* | 1. Document: G8 Dementia Summit Communique | Intergovernmental | 2012 | General Public |
| 8. International Federation on Ageing  <https://www.ifa-fiv.org/>  The goal of IFA *is “to be the global point of connection and networks of experts and expertise to influence and shape age-related policy.”* | 1. Website | Non-Profit | 2020-22 | General Public, Professionals |
| **CANADA**  **(C)** | | | | |
| 1. Dementia Society Ottawa  [www.dementiahelp.ca](http://www.dementiahelp.ca)  *“We provide compassionate support, tailored coaching and practical education for everyone impacted by dementia while building a dementia inclusive community.”* | 1. Website | Non-Profit | 2020-22 | General Public |
| 2. Alzheimer Society of Canada  [www.alzheimer.ca](http://www.alzheimer.ca)  The mission of ASC is *“to alleviate the personal and social consequences of Alzheimer’s and related diseases and to promote the search for causes, treatments and a cure.”* | 1. Website 2. Report: YOD Gap Analysis 3. Report: Support Group Facilitators Guide | Non-Profit | 2020-22  n.d.  2018 | General Public, Professionals |
| 3. Alzheimer Society of Ontario  [www.alzheimer.ca/on](http://www.alzheimer.ca/on)  ASO is *“the province’s leading care and research charity committed to helping people with Alzheimer’s disease and other dementias.”* | 1. Website 2. Blog – Mary Beth Wighton 3. Blog – Lisa Raitt 4. Blog – Robin Barratt 5. Blog – Keith Barratt 6. Blog – After a dementia diagnosis 7. Blog – I am a person with dementia… 8. Blog – At 21 Alzheimer’s is the last thing… 9. Blog – Explaining dementia to kids 10. Blog – Father’s Day | Non-Profit | 2020-22  2017  2019  2019  2019  2018  2018  2017  2016  2019 | General Public, Professionals |
| 4. Alzheimer Society of Sudbury-Manitoulin North Bay and Districts  [www.alzheimer.ca/sudburymanitoulin/en](http://www.alzheimer.ca/sudburymanitoulin/en) | 1. Website | Non-Profit | 2020-22 | General Public |
| 5. Alzheimer Society of Toronto  [www.alz.rto](http://www.alz.rto) | 1. Website | Non-Profit | 2020-22 | General Public |
| 6. Alzheimer Society Waterloo Wellington  [www.alzheimer.ca/ww/en](http://www.alzheimer.ca/ww/en) | 1. Website | Non-Profit | 2020-22 | General Public |
| 7. Alzheimer Society of Durham Region  [www.alzheimer.ca/durham/en](http://www.alzheimer.ca/durham/en) | 1. Website | Non-Profit | 2020-22 | General Public |
| 8. Alzheimer Society Southwest Partners  [www.alzswp.ca](http://www.alzswp.ca) | 1. Website | Non-Profit | 2020-22 | General Public |
| 9. Alzheimer Society Kingston Frontenac Lennox & Addington Counties  [www.alzheimer.ca/kfla/en](http://www.alzheimer.ca/kfla/en) | 1. Website | Non-Profit | 2020-22 | General Public |
| 10. Alzheimer Society York Region  [www.alzheimer.ca/york/en](http://www.alzheimer.ca/york/en) | 1. Website | Non-Profit | 2020-22 | General Public |
| 11. Alzheimer Society Niagara Region  [www.alzheimer.ca/niagara/en](http://www.alzheimer.ca/niagara/en) | 1. Website | Non-Profit | 2020-22 | General Public |
| 12. Public Health Agency of Canada  [www.canada.ca/en/public-health](http://www.canada.ca/en/public-health)  *“The PHAC is part of the federal health portfolio. Its activities focus on preventing disease and injuries, responding to public health threats, promoting good physical and mental health, and providing information to support informed decision making.”* | 1. Report: National Dementia Conference 2. Report: National Dementia Strategy | Government | 2018  2019 | General Public, Professionals |
| 13. Canadian Academy of Health Sciences  [www.cahs-acss.ca](http://www.cahs-acss.ca)  *“The CAHS brings together Canada’s top-ranked health and biomedical scientists and scholars to make a positive impact on the urgent health concerns of Canadians.”* | 1. Report: Improving the Quality of Life and Care | Non-Profit | 2019 | General Public, Professionals |
| 14. Senate Committee on Social Affairs, Science & Technology  [www.sencanada.ca/content/sen/committee/421/SOCI/Reports/SOCI_6thReport_DementiaInCanada-WEB_e.pdf](http://www.sencanada.ca/content/sen/committee/421/SOCI/Reports/SOCI_6thReport_DementiaInCanada-WEB_e.pdf) | 1. Report: National Strategy for Dementia Friendly | Government | 2016 | General Public |
| 15. Government of Canada (Health)  [www.canada.ca/content/dam/canada/health-canada/migration/healthy-canadians/alt/pdf/diseases-conditions-maladies-affections/disease-maladie/dementia-demence/dementia-demence-plan-eng.pdf](http://www.canada.ca/content/dam/canada/health-canada/migration/healthy-canadians/alt/pdf/diseases-conditions-maladies-affections/disease-maladie/dementia-demence/dementia-demence-plan-eng.pdf) | 1. Report: Dementia Research & Prevention Plan | Government | 2014 | General Public, Professionals |
| 16. Canadian Institute for Health Information  [www.cihi.ca](http://www.cihi.ca)  CIHI *“provides comparable and actionable data and information that are used to accelerate improvements in health care, health system performance and population health across Canada*. | 1. Website | Non-Profit | 2020-22 | Professionals |
| 17. Veteran’s Affairs Canada  [www.veterans.gc.ca/eng](http://www.veterans.gc.ca/eng)  *“Veterans Affairs Canada’s mandate is to support the well-being of veterans and their families, and to promote recognition and remembrance of the achievements and sacrifice of those who served Canada in times of war, military conflict and peace.”* | 1. Website 2. Report: Dementia Care Evaluation | Government | 2020-22  2009 | General Public, Professionals  General Public, Professionals |
| 18. Ontario Ministry of Health and Long Term Care  [www.ontario.ca/page/developing-ontarios-dementia-strategy-discussion-paper](http://www.ontario.ca/page/developing-ontarios-dementia-strategy-discussion-paper) | 1. Report: Ontario’s Dementia Strategy Discussion Paper | Government | 2016 | General Public |
| 19. Health Quality Ontario  Health Quality Ontario  [www.hqontario.ca](http://www.hqontario.ca)  *“Health Quality Ontario is the provincial lead on the quality of health care. We help nurses, doctors and other health care professionals working hard on the frontlines be more effective in what they do – by providing objective advice and data, and by supporting them and government in improving health care for the people of Ontario.”* | 1. Report: Dementia Care for People Living in the Community | Government | 2018 | Professionals |
| 20. YouQuest  [www.youquest.ca](http://www.youquest.ca)  *“YouQuest improves quality of life for people with young-onset dementia and their care partners. YouQuest is a registered charity addressing a void for this underserved community in Canada.”* | 1. Website 2. Report: Pilot Evaluation | Non-Profit | 2020-22  2020 | General Public |
| 21. Dementia Dialogue  [www.dementiadialogue.ca](http://www.dementiadialogue.ca)  *“Our podcast provides people with lived experience a way to share their stories with each other and the broader community. Listeners who have dementia, care partners, and others gain insight and strengthen their adaptive skills. Episodes also help the broader community understand what it means to live with dementia and how they can support people.”* | 1. Website 2. Podcast Season 1 Episode 1 3. Podcast Season 1 Episode 2 4. Podcast Season 1 Episode 3 5. Podcast Season 1 Episode 4 6. Podcast Season 1 Episode 5 | Education | 2020-22  n.d.  n.d.  n.d.  n.d.  n.d. | General Public, Professionals |
| 22. Dementia Advocacy Canada  [www.dementiacanada.com](http://www.dementiacanada.com)  *“We are a grassroots group of people living with dementia and care partners. We want to influence policy, inform program development and improve access to support and services across Canada.”* | 1. Website | Grassroots | 2020-22 | General Public |
| 23. National Institute for the Care of the Elderly  [www.nicenet.ca](http://www.nicenet.ca)  *“NICE is an international network of researchers, practitioners and students dedicated to improving the care of older adults, both in Canada and abroad.”* | 1. Website | University | 2020-22 | Professionals |
| 24. Ontario Dementia Advisory Group  [www.odag.ca](http://www.odag.ca)  *“ODAG is a group of individuals with dementia living in Ontario.”* | 1. Website | Grassroots | 2020-22 | General Public |
| 25. Murray Alzheimer Research & Education Program  [www.the-ria.ca/murray-alzheimer-research-education-program-marep](http://www.the-ria.ca/murray-alzheimer-research-education-program-marep)  MAREP *“is a collaborative research and education program that aims to enhance well-being for individuals impacted by dementia, including people living with dementia, care partners, health care providers and community members.”* | 1. Website 2. Video series: A new voice: Living well 3. Report: Young Onset Dementia Inspirational Guide | University | 2020-22  n.d.  2018 | General Public, Professionals |
| 26. Canadian Home Care Association  [www.cdnhomecare.ca](http://www.cdnhomecare.ca)  *“The CHCA is a national non-profit membership association dedicated to advancing excellence in home and community care.”* | 1. Website 2. Report: Informing National Dementia Strategy | Non-Profit | 2020-22  2018 | General Public, Professionals |
| 27. Canadian Nurses Association  [www.cna-aiic.ca/en/home](http://www.cna-aiic.ca/en/home)  *“As the national voice of nursing in Canada, we: Act in the public interest for Canadian nursing and nurses, providing national and international leadership in nursing and health; Advocate for publicly funded, no-for-profit health system; Advance nursing excellence and positive health outcomes; Promote profession-led regulation.”* | 1. Website 2. Report: Dementia in Canada | Non-Profit | 2020-22  2016 | Professionals |
| 28. College of Family Physicians of Canada  [www.cfpc.ca/en/home](http://www.cfpc.ca/en/home)  *“The College establishes the standards for and accredits postgraduate family medicine training in Canada’s 17 medical schools. It reviews and certifies continuing professional development programs and materials that enable family physicians to meet certification and licensing requirements. The CFPC provides high-quality services, supports family medicine teaching and research, and advocates on behalf of the specialty of family medicine, family physicians and the patients they serve.”* | 1. Website | Non-Profit | 2020-22 | Professionals |
| 29. Canadian Geriatric Society  [www.thecanadiangeriatricssociety.wildapricot.org](http://www.thecanadiangeriatricssociety.wildapricot.org)  *“Become part of a vibrant national organization that is addressing THE defining health care challenge of our generation – the provision of compassionate, effective and sustainable health care for the rapidly growing number of older Canadians.”* | 1. Website | Non-Profit | 2020-22 | Professionals |
| 30. Toronto Dementia Network  [www.tdn.alz.to](http://www.tdn.alz.to)  *“The number one source for dementia related services.”* | 1. Website | Non-Profit | 2020-22 | Professionals |
| 31. Canadian Consortium on Neurodegeneration in Aging  [www.ccna-ccnv.ca](http://www.ccna-ccnv.ca)  *“The CCNA provides the infrastructure and support that facilitates collaboration amongst Canada’s top dementia researchers and clinicians. CCNA is the premier hub for Alzheimer’s disease and dementia research in Canada, and a global leader in research on all aspects of neurodegenerative disease from prevention to treatment to improving the quality of life of those living with the disease.”* | 1. Website | Government | 2020-22 | Professionals |
| 32. Centre for Aging & Brain Health Innovation (Baycrest)  [www.cabhi.com](http://www.cabhi.com)  *“CABHI, powered by Baycrest, helps innovators develop, disseminate, scale, and promote adoption of promising innovations in the aging and brain health sector.”* | 1. Website | Non-Profit,  Government &  Industry | 2020-22 | General Public, Professionals |
| 33. Indigenous Cognition & Aging Awareness Research Exchange  [www.i-caare.ca](http://www.i-caare.ca) | 1. Website 2. Report: What is Dementia Indigenous Perspectives 3. Report: Signs & Symptoms 4. Report: Preventing Dementia 5. Report: What to Expect after Diagnosis 6. Report: Path of Dementia | University | 2020-22  2015  2015  2017  2017  2017 | General Public, Professionals |
| 34. McMaster i-GeriCare  [iGeriCare \| Bringing Clarity to Dementia (healthhq.ca)](https://igericare.healthhq.ca/en)  *“A diagnosis of dementia can be challenging for patients, families and caregivers. Our easy to understand lessons, helpful resources and online community will help reduce stress and increase your quality of life.”* | 1. Website | University | 2020-22 | Professionals |
| 35. Weston Brain Institute  [www.westonfoundation.ca/weston-brain-institute](http://www.westonfoundation.ca/weston-brain-institute)  *“Reducing the incidence and progression of neurodegenerative diseases to enable healthy aging for Canadians.”* | 1. Website | Non-Profit | 2020-22 | Professionals |
| 36. Women’s Brain Health  [www.womensbrainhealth.org](http://www.womensbrainhealth.org)  *“Women’s Brain Health Initiative is a Canadian and U.S. charitable foundation established in 2012 and is solely dedicated to protecting the brain health of women.”* | 1. Website | Non-Profit | 2020-22 | Professionals |
| 37. Reitman Centre  [www.mountsinai.on.ca/care/psych/patient-programs/geriatric-psychiatry/dementia-support/caregivers-and-family-members-caring-for-someone-dementia/carers/reitman-centre-carers-program](http://www.mountsinai.on.ca/care/psych/patient-programs/geriatric-psychiatry/dementia-support/caregivers-and-family-members-caring-for-someone-dementia/carers/reitman-centre-carers-program)  *“The CARERS (Coaching, Advocacy, Respite, Education, Relationship, Simulation) Program is an 8 session skills-based group for family caregivers. The focus in on practical skills based tools and emotional supports needed to provide care for family members with dementia.”* | 1. Website | Government | 2020-22 | General Public, Professionals |
| 38. Dementia Connections  [www.dementiaconnections.ca](http://www.dementiaconnections.ca)  *“…we are now a sole purpose company on a mission to amplify the voices of lived experience and share expert advice about living well with dementia.”* | 1. Website | For Profit | 2020-22 | General Public |
| 39. Parkinson Canada  [www.parkinson.ca](http://www.parkinson.ca)  *“…research, provides care, and creates resources, news and events that transform the lives of people touched by Parkinson’s.”* | 1. Website | Non-Profit | 2020-22 | General Public, Professionals |
| 40. Huntington Society of Canada  [www.huntingtonsociety.ca](http://www.huntingtonsociety.ca)  *“To improve the quality of life of those affected by Huntington disease. We will address our mission through the oversight and facilitation of excellent support services, providing access to the best and most up-to-date educational resources, increasing national and global awareness, advocacy and investing in promising research.”* | 1. Website 2. Report: Advanced Stage 3. Report: Carers Guide 4. Report: Understanding Behaviour 5. Report: Loss and Grief 6. Report: Physicians Guide   Report: A Resource for Families | Non-Profit | 2020-22  n.d.  2018  2016  2004  2013  2008 | General Public, Professionals |
| 41. Association for Frontotemporal Dementia^[[3]](#footnote-3)^  [www.theaftd.org](http://www.theaftd.org)  *“AFTD’s mission is to improve the quality of life of people affected by FTD and drive research to a cure.”* | 1. Website 2. Report: Managing a New Diagnosis 3. Report: What about the kids 4. Report: Talking with children and teens 5. Film: It is what it is | Non-Profit | 2020-22  2013  2012  2011  n.d. | General Public, Professionals |
| 42. St. Joseph Health Centre  [www.sjhcg.ca](http://www.sjhcg.ca)  *“We are Guelph’s leading, full-accredited, not-for-profit provider of resident long term care, complex medical, and rehabilitation services. We also offer excellent community support services for people who live in their own homes but need a little assistance in their day-to-day lives.”* | 1. Website | Non-Profit | 2020-22 | General Public |
| 43. Canadian Organization for Rare Disorders  [www.raredisorders.ca](http://www.raredisorders.ca)  *“CORD is Canada’s national network for organizations representing all those with rare disorders.”* CORD’s mission is to *“provide a strong common voice to advocate for health policy and a healthcare systems that works for those with rare disorders.”* | 1. Website | Non-Profit | 2020-22 | General Public, Professionals |
| 44. Ontario Neurodegenerative Disease Research Initiative  [www.ondri.ca](http://www.ondri.ca)  *“ONDRI’s four aims represent areas of research focused on collecting, analyzing, and validating data from people living with diseases that can cause cognitive impairment and dementia. ONDRI takes a unique multi-dimensional approach to its research, with a cross-disease, cross-platform perspective that allows new types of questions to be asked.”* | 1. Website | Non-Profit | 2020-22 | General Public, Professionals |
| 45. Lewy Body Dementia Canada  [www.lewybodydementia.ca](http://www.lewybodydementia.ca)  “*Find the best, easy to understand, reliable and useful information about Lewy Body Dementia right here, including the most important information, symptoms, treatments and insights to make the best of a very challenging condition.”* | 1. Website | Grassroots | 2020-22 | General Public |
| 46. Alzheimer Group Inc.  [www.agiteam.org](http://www.agiteam.org)  AGI *“is a charitable organization that offers therapeutic programs to individuals living with Alzheimer’s disease and other dementias. Additionally, AGI provides support services to families and professional care partners, focusing on best practices in dementia care, while sensitizing the community at large through education and awareness.”* | 1. Website | Non-Profit | 2020-22 | General Public, Professionals |
| 47. Anishinaabek Dementia Care  [www.anishinaabekdementiacare.ca](http://www.anishinaabekdementiacare.ca)  *“The goal of this project is to build sustainable community-owned and directed strategies to support health aging and dementia care in Indigenous communities, with a focus on cultural safety and trauma-informed care.”* | 1. Website 2. Film: Anishinaabek Dementia Care | Government | 2020-22 | General Public, Professionals |
| 48. Health Care Excellence Canada  [Healthcare Excellence Canada](https://healthcareexcellence.ca/en)  *“Healthcare Excellence Canada works with partners to spread innovations, build capability and catalyze policy changes so that everyone in Canada has safe and high-quality healthcare.”* | 1. Website | Government | 2020-22 | Professionals |
| 49. Behavioural Supports Ontario  [Home - behaviouralsupportsontario.ca](https://behaviouralsupportsontario.ca/)  *“The BSO initiative was created to enhance health care services for older adults in Ontario with complex and responsive behaviours associated with dementia, mental health, substance use and/or other neurological conditions. The initiative also provides enhanced family caregiver support in the community, in long term care or wherever the patient and/or caregiver(s) reside.”* | 1. Website | Government | 2020-22 | Professionals |
| 50. MINT Memory Clinics  [www.mintmemory.ca](http://www.mintmemory.ca)  *“Everyday, people living with dementia and other memory disorders go undiagnosed or under-treated, placing avoidable strain on individuals and their families. MINT Memory Clinics are helping to close these gaps by offering timely access to compassionate memory care and navigational supports, all in one place that’s close to home.”* | 1. Website | Government | 2020-22 | General Public, Professionals |
| 51. Early Onset Dementia Alberta Foundation  [www.eodaf.ca](http://www.eodaf.ca)  *“EODAF strives to build awareness and promote culture change for persons living with dementia under the age of 65.”* | 1. Website | Non-Profit | 2020-22 | General Public |
| 52. RaDAR  [www. cchsa-ccssma.usask.ca/ruraldementiacare](https://cchsa-ccssma.usask.ca/ruraldementiacare/)  *“…are facing the challenge to improve the delivery of rural dementia care across the continuum: from early detection and diagnosis to end-of-life care.”* | 1. Website | University, Government | 2020-22 | General Public, Professionals |
| 53. Champlain Community Support Network  [www.ccsn-rscc.org](http://www.ccsn-rscc.org)  *“CCSN represents more than 40 non-profit health service providers across Eastern Ontario. Our bilingual network works to create a community where seniors and adults living with disabilities can live independently, safely, and comfortably at home.”* | 1. Report: People with YOD and their Families | Non-Profit | 2019 | Professionals |
| **England**  **(E)** | | | | |
| 1. Alzheimer’s Society  [www.alzheimers.org.uk](http://www.alzheimers.org.uk)  *“We’re a vital source of support and a powerful force for change for everyone affected by dementia.”* | 1. Website | Non-Profit | 2020-22 | General Public, Professionals |
| 2. National Health Service England  [www.england.nhs.uk](http://www.england.nhs.uk) | 1. Website 2. Report Implementation Guide & Resource Pack 3. Report Dementia Good Care Planning | Government | 2020-22  2017  2017  n.d. | General Public, Professionals |
| 3. Department of Health  [www.gov.uk/government/organisations/department-of-health-and-social-care](http://www.gov.uk/government/organisations/department-of-health-and-social-care) | 1. Report PMs Challenge on Dementia 2. Report PMs Challenge Implementation 3. Report Implementation Road Map 4. iv. Report Dementia Strategy | Government | 2015  2016  2016  2009 | General Public |
| 4.National Institute for Clinical Excellence  [www.nice.org.uk](http://www.nice.org.uk)  The National Institute for Health and Care Excellence provides *“national guidance and advice to improve health and care.”* | 1. Report: Assessment, Management & Support | Government | 2018 | Professionals |
| 5. Public Health England  [www.gov.uk/government/organisations/public-health-england](http://www.gov.uk/government/organisations/public-health-england)  *“PHE was an executive agency of the Department of Health and Social Care in England…to protect and improve health and wellbeing and reduce health inequalities.”* Its current functions are now delivered by the UK Health Security Agency and Office for Health Improvement and Disparities. | 1. Website | Government | 2018 | General Public, Professionals |
| 6. Wellcome Trust  [www.wellcome.org](http://www.wellcome.org)  *“Wellcome is a global charitable foundation. We want everyone to benefit from science’s potential to improve health and save lives.”* | 1. Website | Non-Profit | 2020-22 | General Public |
| 7. National Dementia Action Alliance  [www.dementiaaction.org.uk](http://www.dementiaaction.org.uk)  Since April 2020 known as Local Dementia Alliance *“we are the alliance for organisations across England to connect, take action on dementia and work together to build Dementia Friendly Communities.”* | 1. Website | Non-Profit | 2020-22 | General Public, Professionals |
| 8. Cornwall National Health Service Trust  [www.cornwallft.nhs.uk](http://www.cornwallft.nhs.uk) | 1. Website | Government | 2020-22 | General Public |
| 9. Cumbria, Northumberland, Tyne & Wear National Health Service Trust  [www.cntw.nhs.uk](http://www.cntw.nhs.uk) | 1. Website | Government | 2020-22 | General Public |
| 10. Brighton and Sussex University Hospitals National Health Service Trust  [www.bsuh.nhs.uk](http://www.bsuh.nhs.uk) | 1. Website | Government | 2020-22 | General Public |
| 11. Kent County Council  [www.kent.gov.uk](http://www.kent.gov.uk) | 1. Website | Government | 2020-22 | General Public |
| 12. Nottingham City Council  [www.nottinghamcity.gov.uk](http://www.nottinghamcity.gov.uk) | 1. Website | Government | 2020-22 | General Public |
| **UNITED KINGDOM**  **(UK)** |  |  |  |  |
| 1. National Health Service UK  [www.nhs.uk](http://www.nhs.uk) | 1. Website | Government | 2020-22 | General Public |
| 2. National Collaborating Centre for Mental Health  [www.rcpsych.ac.uk/improving-care/nccmh](http://www.rcpsych.ac.uk/improving-care/nccmh)  *“The NCCMH reviews the evidence and co-produces guidance, standards, workforce competencies and quality improvement initiatives to enable the delivery of high-quality, equitable mental health care.”* | 1. Report: Dementia Care Pathway | Professional | 2018 | General Public, Professionals |
| 3. Dementia UK  [www.dementiauk.org](http://www.dementiauk.org)  *“Our vision is provide the specialist and compassionate support for all families that it through our Admiral Nurse service.”* | 1. Website | Non-Profit | 2020-22 | General Public, Professionals |
| 4. Royal College of General Practitioners  [www.rcgp.org.uk](http://www.rcgp.org.uk)  *“We are the professional membership body for family doctors in the UK and overseas. We are committed to improving patient care, clinical standards and GP training.”* | 1. Website | Professional | 2020-22 | Professionals |
| 5. UK Research and Innovation  [www.ukri.org](http://www.ukri.org)  *“UKRI is the national funding agency investing in science and research in the UK, bringing together the seven research councils, Innovate UK and Research England.”* | 1. Website | Non-governmental Public Body | 2020-22 | Professionals |
| 6. Social Care Institute for Excellence  [www.scie.org.uk](http://www.scie.org.uk)  “***SCIE improves the lives of people of all ages by co-producing, sharing, and supporting the use of the best available knowledge and evidence about what works in practice.”*** | 1. Website | Non-Profit | 2020-22 | Professionals |
| 7. Alzheimer Research UK  [www.alzheimersresearchuk.org](http://www.alzheimersresearchuk.org)  *“We are the leading research dementia and Alzheimer’s disease research charity.”* | 1. Website | Non-Profit | 2020-22 | General Public, Professionals |
| 8. Age UK  [www.ageuk.org.uk](http://www.ageuk.org.uk)  *“Age UK is the leading charity for older people.”* | 1. Website 2. Report: Dementia & Cognitive Decline | Non-Profit | 2020-22  2014 | General Public, Professionals |
| 9. DEEP  [www.dementiavoices.org.uk](http://www.dementiavoices.org.uk)  *Dementia Engagement and Empowerment project is “the UK network of dementia voices. DEEP consists of around 80 groups of people with dementia – groups that want to change things.”* | 1. Website | Non-Profit | 2020-22 | General Public |
| 10. House of Commons  [www.parliament.uk/business/commons](http://www.parliament.uk/business/commons) | 1. Report: Dementia Policy, Services, Statistics | Government | 2019 | General Public |
| 11. Rare Dementia Support  [www.raredementiasupport.org](http://www.raredementiasupport.org)  *”…is a world-leading, UK-based service provided by the UCL Dementia Research Centre and partners and funded by The National Brain Appeal. The RDS Team is a large team comprised of international clinical and non-clinical specialists and people affected by rare dementias, collectively working together to bring their skills and expertise in dementia research, education and support.”* | 1. Website | Non-Profit, University, Government | 2020-22 | General Public, Professionals |
| **WALES**  **(W)** | | | | |
| 1. Alzheimer’s Society Cymru (Wales)  [www.alzheimers.org.uk/about-us/wales](http://www.alzheimers.org.uk/about-us/wales)  *“At Alzheimer’s Society we’re working towards a world where dementia no longer devastates lives. We do this by giving help to those living with dementia today, and providing hope for the future.”* | 1. Website | Non-Profit | 2020-22 | General Public |
| 2. Welsh Government  [www.gov.wales](http://www.gov.wales) | 1. Report: Dementia Action Plan | Government | 2018 | General Public |
| 3. National Health Service Wales  [www.wales.nhs.uk](http://www.wales.nhs.uk) | 1. Website 2. Report: Evidence-based Psychological Therapy | Government | 2020-22  2017 | General Public, Professionals |
| 4. Social Care Wales  [www.socialcare.wales](http://www.socialcare.wales) | 1. Website | Government | 2020-22 | Professionals |
| 5. Older People’s Commissioner for Wales  [www.olderpeople.wales](http://www.olderpeople.wales)  *“An independent voice and champion for older people. The Commissioner is working for a Wales where older people are valued, rights are upheld and no-one is left behind.”* | 1. Website | Government | 2020-22 | General Public |
| 6. Carer’s Trust  [www.carers.org](http://www.carers.org)  *“Our vision at Carers trust is that unpaid carers are heard, values and have access to the support, advice and resources they need to live a fulfilling life alongside caring.”* | 1. Website | Non-Profit | 2020-22 | General Public |
| 7. TIDE  [www.tide.uk.net](http://www.tide.uk.net)  *“The voice, friend and future of all carers of people with dementia.”* | 1. Website | Non-Profit | 2020-22 | General Public, Professionals |
| 8. Health and Care Research Wales  [www.healthandcareresearchwales.org](http://www.healthandcareresearchwales.org)  *“Health and Care Research Wales is a networked organization supported by the Welsh Government which brings together a wide range of partners across the NHS in Wales, local authorities, universities, research institutions, third sector and others.”* | 1. Website | Government | 2020-22 | General Public, Professionals |
| 9. Ageing & Dementia @ Bangor  [www.dsdc.bangor.ac.uk/supporting-people.php.en](http://www.dsdc.bangor.ac.uk/supporting-people.php.en)  *“Our remit is to address the challenges and opportunities posed by demographic change and an ageing society and to: promote the health, quality of life and well-being of older people, people with dementia and their supports; improve the quality of care and services; inform policy and practice development; ensure the patient and public voice is at the health of our work.”* | 1. Website | University | 2020-22 | General Public, Professionals |
| 10. Rural Health & Care Wales  [www.ruralhealthandcarewales.org](http://www.ruralhealthandcarewales.org)  *“RHCW is an organization of excellence that is leading the way in the field of rural health and social care in Wales, the UK and internationally.”* | 1. Website | Government | 2020-22 | General Public |
| 11. ENRICH Cymru  <https://www.walessscr.org/en/enrich-cymru>  *“ENRICH Cymru is a network is co-hosted by the Wales School for Social Care Research and the Centre for Ageing and Dementia Research at Swansea University.”* | 1. Website | University | 2020-22 | General Public, Professionals |
| 12. Age Cymru  [www.ageuk.org.uk/cymru](http://www.ageuk.org.uk/cymru)  *“We provide vital services directly to older people in the community and we meet the highest standards so you can be assured of consistency and quality wherever you see the Age Cymru name and logo.”* | 1. Report: Dementia and Cognitive Decline | Non-Profit | 2014 | General Public |
| 13. Dewis Cymru  [www.dewis.wales](http://www.dewis.wales)  *“This is a search service allowing people to look for support and information across Wales and in the UK. It provides links to other websites.”* | 1. Website | Government | 2020-22 | General Public |
| 14. Wales School for Social Care Research  [www.walessscr.org](http://www.walessscr.org)  *“The School was created to improve and increase social care research in Wales.”* | 1. Website | Government | 2020-22 | Professionals |

1. All documents and website were accessed during the period of June 2020 – January 2022. Documents embedded in the website were also reviewed. [↑](#footnote-ref-1)
2. Document not dated. [↑](#footnote-ref-2)
3. The AFD is an American organization and also provides supports to people living in Canada. [↑](#footnote-ref-3)
