# Supplementary material for: Situating support for people living with rarer forms of dementia
Source: BMC Geriatr. 2023 Oct 6;23:627. doi: 10.1186/s12877-023-04268-4 (PMC10557369; doi:10.1186/s12877-023-04268-4)
Supplement: Supplementary file 4 — Additional File 4: Table S1: Sample characteristics for Blinded (UK) practitioner members (N = 62). Table S2: Sample characteristics for Canadian practitioners (N = 46). Table S3: Frequency of work with people affected by rare dementias [file 12877_2023_4268_MOESM4_ESM.docx]

Additional File 4

Table S1: Sample characteristics for *Blinded* (UK) practitioner members (N = 62)

| Variables | n (%) |
| --- | --- |
| Location |  |
| England | 48 (77.42) |
| Scotland | 2 (3.23) |
| Wales | 5 (8.06) |
| Europe | 4 (6.45) |
| North America | 2 (3.23) |
| Hong Kong | 1 (1.61) |
| Sector^a^ |  |
| Health care | 36 (55.38) |
| Social care | 4 (6.15) |
| Non-profit | 18 (27.69) |
| Private business | 7 (10.77) |
| Profession |  |
| Nurse | 15 (24.19) |
| Occupational Therapist | 6 (9.68) |
| Speech & Language Therapist | 9 (14.52) |
| Social Worker | 4 (6.45) |
| Dementia Support Worker | 4 (6.45) |
| Dementia Advisor/Coordinator | 10 (16.13) |
| GP | 2 (3.23) |
| Physiotherapist | 2 (3.23) |
| Psychologist | 6 (9.68) |
| Neurologist | 1 (1.61) |
| Student | 1 (1.61) |
| Non-health professions | 2 (3.23) |
| ^a^ n=65, due to multiple responses |  |

Table S2: Sample characteristics for Canadian practitioners (N = 46)

| Variables | n (%) |
| --- | --- |
| Location |  |
| Ontario | 45 (97.83) |
| Alberta | 1 (2.17) |
| Sector^a^ |  |
| Health care | 11 (28.20) |
| Non-profit | 25 (64.10) |
| Long Term Care | 2 (5.13) |
| Higher Education | 1 (2.56) |
| Profession^b^ |  |
| Nurse | 2 (5.56) |
| Occupational Therapist | 1 (2.78) |
| Speech & Language Therapist/CDAs | 3 (8.33) |
| Social Worker | 19 (52.78) |
| Personal Support Worker | 2 (5.56) |
| Recreation Therapist | 4 (11.11) |
| Researcher | 1 (2.78) |
| Other | 4 (11.11) |
| ^a^ n=39, due to multiple responses. ^b^ n=36 |  |

Table S3: Frequency of work with people affected by rare dementias

| Frequency | *Blinded* Members ^a^ | | Canadian Practitioners | |
| --- | --- | --- | --- | --- |
|  | PLWrD n (%) | Carer n (%) | PLWrD ^b^ n (%) | Carer ^c^ n (%) |
| Never before | 1 (1.92) | 3 (5.77) | 2 (5.71) | 1 (2.94) |
| Once | 1 (1.92) | 0 (0.00) | 1 (2.86) | 1 (2.94) |
| Occasionally | 22 (42.31) | 19 (36.54) | 16 (45.71) | 17 (50.00) |
| Regularly | 28 (53.85) | 30 (57.69) | 16 (45.71) | 15 (44.12) |

^a^ n=52. ^b^ n=35. ^c^ n=34
